# Ethical issues related to consent for intrapartum trials

**DOI:** 10.1186/s12978-017-0426-y

**Published:** 2017-12-14

**Authors:** Hema Dhumale, Shivaprasad Goudar

**Affiliations:** 1 KLE University’s J N Medical College, Nehru Nagar, Belagavi, 590010 India; 2 JNMC- UMKC Women’s and Children’s Health Research Unit, KLE University’s J N Medical College, Nehru Nagar, Belagavi, 590010 India

**Keywords:** Intrapartum clinical trial, Informed consent, Ethical issues

## Abstract

Informed consent is the heart of ethical research. For any consent to be ethically valid, it should meet certain critical criteria— disclosure and understanding of relevant information, decision making competency of the participants, voluntariness of the decision and documentation of the agreement. Meeting all these criteria to obtain ethically valid consent from laboring women while conducting intrapartum trials is challenging because there is little time available during labor to provide study specific information necessary for the participant to understand and decide to sign the consent form. Moreover, women during labor may be anxious and distressed due to labor pains which is thought to interfere with the capacity to make decisions in some cases. Emphasis on these concerns may ultimately lead to the exclusion of many eligible women in labor from intrapartum clinical trials. In this paper, we discuss the ethical challenges and also the proposed recommendations to obtain ethically valid consent from women for conducting intrapartum clinical trials.

## Case background

Postpartum hemorrhage (PPH) is defined as blood loss of 500 ml or more within 24 h of delivery. Blood loss of more than 1000 ml is considered severe PPH. Atonic PPH is the most common cause of maternal mortality and morbidity in low income countries, particularly in Africa and Asia where PPH contributes to 30% of maternal deaths [1]. Maternal mortality and morbidity due to atonic PPH can be prevented by the use of prophylactic uterotonic agents during the active management of the third stage of labor. Though oxytocin injection is the ideal uterotonic for this purpose, the requirement of strict cold storage for maintaining its efficacy prevents it from being used in many low- and middle-income tropical country settings. Carbetocin room temperature stable (RTS) has been considered to be a promising intervention for reducing PPH in settings where cold storage is difficult to maintain.

A recent Phase III trial aims to evaluate the effectiveness of carbetocin RTS 100 μg intramuscular (IM) compared to oxytocin 10 IU,IM in preventing PPH in vaginal deliveries [2]. Women with singleton pregnancy expecting to deliver vaginally were approached early in labor (≤6 cm of cervical dilatation) for participation and written informed consent was taken. The main objective of this trial is to determine if carbetocin RTS is similar in efficacy to oxytocin in preventing PPH.

The entire consenting process was audio-visual (A-V) recorded and only eligible women who consented to A-V recording were recruited in to the trial. The issue of A-V recording the informed consent process is unique and applicable only in India. In 2015, the Drug Controller General of India (DCGI) amended the earlier regulation and made A-V recording of the informed consent process mandatory for trials involving vulnerable population and trials related to new drugs [3].

All eligible consented women were randomly assigned to receive either a single dose of oxytocin 10 IU,IM or a single dose of carbetocin RTS 100 μg,IM at the second stage of labor when vaginal delivery was imminent. Placental delivery in all women was conducted by controlled cord traction immediately after cord clamping and blood loss was measured using the BRASS-V blood collection drape for 1 h following delivery.

## Ethical discussion

Obtaining ethically valid consent from laboring women while conducting intrapartum trials is challenging because there is little time available during labor to provide study specific information necessary for the participant to understand and decide to sign the consent form. Moreover, women during labor may be anxious and distressed due to labor pains which is thought to interfere with the capacity to make decisions in some cases. Emphasis on these concerns may ultimately lead to the exclusion of many eligible women in labor from intrapartum clinical trials.

The two main ethical issues regarding the consent process for intrapartum trials addressed in this case study are:

1. Exclusion of women in the active stage of labor with cervical dilatation of more than 6 cm

Women in the active stage of labor with cervical dilation of more than 6 cm were excluded on the grounds that women would be too distressed due to labor pains to provide informed written consent. The ability of a woman in labor to understand new information and to make an informed decision varies widely. The nature of the intrapartum complication being studied in the trial also determines the time available for providing informed consent. Despite arguments questioning the competency of laboring women to give informed written consent late in labor, there is evidence that most anticipated variables—e.g. labor pains, duration of labor, anxiety, and opioid analgesics—may not interfere with the ability of women in labor to understand the information provided to them and make decisions [4]. Many women with these conditions are still capable of giving their own consent: it should not be assumed that they lack capacity. Hence denying women in labor from inclusion in the trial based only on the cervical dilatation cutoff of ≤6 cm (early labor) is scientifically and ethically incorrect.

There is also a recommendation in the literature to consider the obstetric care provider (doctor/midwife) as the “gatekeeper” to assess the physical and emotional state of the laboring woman. The pregnant woman’s competency depends upon many variables and this recommendation allows for determination of her ability to provide consent on an individual basis [5]. Allowing the healthcare provider to act as a gatekeeper could be a novel alternative approach.

2. A-V recording of consent process for intrapartum clinical trials in India

As per the DCGI regulation A-V recording of consent is mandatory only for trials involving vulnerable populations and trials related to new drugs. It has not been determined whether pregnant women constitute a vulnerable population in India [6]. However, in the present study, A-V recording of the consent process was done because the clinical trial involved carbetocin which is a new drug in India. A-V recording might add to the anxiety and distress of laboring women and may also make them feel vulnerable with respect to maintaining privacy and confidentiality. This could discourage women from participating in intrapartum clinical trials.

## Conclusions

There is a need to develop a standard outline of the intrapartum consent process with optional elements that can be adjusted depending upon the type of the trial and the participants. We propose the following recommendations:

1. Intrapartum women who have received the relevant trial information and signed the informed consent antenatally should be eligible to reconfirm and sign the consent during any stage of labor as long as they remain eligible and competent to provide consent. In acute circumstances, such women may also be allowed to provide oral consent at the time of complication supplemented by signing the written consent at a later stage [7, 8].
2. Intrapartum women who have not received the trial information antenatally should be eligible to sign informed consent in early labor (≤6 cm of cervical dilatation). Such women may still be allowed to sign informed consent even late in labor (≥6 cm of cervical dilatation), provided they are considered competent to provide consent by the obstetric care provider (doctor/midwife), taking into account their individual physical and emotional status.
3. There should be a waiver for A-V recording of the consent process for all intrapartum trials keeping in mind the socio cultural factors prevailing in India and also the need to protect the privacy of laboring women.

## Abbreviations

A-V: Audio-visual
DCGI: Drug Controller General of India
IM: Intramuscular
PPH: Postpartum hemorrhage
RTS: Room temperature stable
